# Patient-rated suitability of a novel electronic device for self-injection of subcutaneous interferon beta-1a in relapsing multiple sclerosis: an international, single-arm, multicentre, Phase IIIb study

**DOI:** 10.1186/1471-2377-10-28

**Published:** 2010-04-30

**Authors:** Virginia Devonshire, Txomin Arbizu, Björn Borre, Michael Lang, Alessandra Lugaresi, Barry Singer, Elisabetta Verdun di Cantogno, Peter Cornelisse

**Affiliations:** 1 Department of Neurology, University of British Columbia, 2211 Wesbrook Mall, V6T 2B5 Vancouver, BC, Canada; 2 Hospital Universitario de Bellvitge Servicio de Neurología, Unitat d'Esclerosi Múltiple, L'Hospitalet de Llobergat, Avenida Gran Via, 08907 Barcelona, Spain; 3 Neurokliniken, Drottninggatan 17, SE-25221 Helsingborg, Sweden; 4 NeuroPoint Patient Academy and Neurological Practice, Pfauengasse 8, 89073 Ulm, Germany; 5 MS Centre, Centro Sclerosi Multipla (VII Livello, Corpo A), Ospedale Clinicizzato "Santissima Annunziata", Via dei Vestini, 66100 Chieti, Italy; 6 The MS Center for Innovations in Care, Missouri Baptist Medical Center, 3009 North Ballas Road, Building B, Suite 207, Saint Louis, MO, 63131, USA; 7 Merck Serono S.A. - Geneva, 9 Chemin des Mines, CH-1202 Geneva, Switzerland (an affiliate of Merck KGaA), Darmstadt, Germany

## Abstract

**Background:**

Multiple sclerosis (MS) currently requires long-term treatment with disease-modifying drugs, administered parenterally up to once daily. The need for regular self-injection can be a barrier to treatment for many patients. Autoinjectors can help patients overcome problems or concerns with self-injection and could, therefore, improve treatment adherence. This study was performed to assess the suitability of a new electronic device for the subcutaneous (sc) administration of interferon (IFN) beta-1a, 44 mcg three times weekly, for relapsing MS.

**Methods:**

In this Phase IIIb, multicentre, single-arm study, patients with relapsing MS who had been consistently self-injecting sc IFN beta-1a using an autoinjector for at least 6 weeks were taught to use the new device and self-administered treatment for 12 weeks thereafter. Patient-rated suitability of the device was assessed at the end of Week 12 using the Patient User Trial Questionnaire. Patient satisfaction with, and evaluation of, the injection process was assessed using the MS Treatment Concern Questionnaire. Trainers evaluated the device using the Trainer User Trial Questionnaire.

**Results:**

At Week 12, 71.6% (73/102) of patients considered the device 'very suitable' or 'suitable' for self-injection; 92.2% (94/102) reported some degree of suitability and only 7.8% (8/102) found the device 'not at all suitable'. At Weeks 4, 8 and 12, most patients reported that injection preparation and clean-up, performing injections and ease of device use in the previous 4 weeks compared favourably with, or was equivalent to, their previous experience of self-injection. Injection-related pain, injection reactions and 'flu-like' symptoms remained stable over the 12 weeks. Each device feature was rated 'very useful' or 'useful' by at least 80% of patients. All trainers and 95.2% (99/104) of patients found device functions 'very easy' or 'easy' to use. Overall convenience was considered the most important benefit of the device.

**Conclusions:**

Most patients considered the new electronic injection device suitable for the sc injection of IFN beta-1a. They found the device easy to use with useful features, and reported benefits such as overall convenience. The device may, therefore, increase treatment adherence in patients with MS, particularly those with injection-related issues.

**Trial registration:**

NCT00735007

## Background

Multiple sclerosis (MS) is a chronic, inflammatory and degenerative disease of the central nervous system that can cause a wide range of debilitating symptoms affecting both mental and physical functions [1]. Although MS remains incurable, treatment with disease-modifying drugs (DMDs) such as interferon (IFN) beta-1a, IFN beta-1b and glatiramer acetate can reduce the frequency of disease exacerbations and may delay disability progression [2-5]

Currently, all approved first-line DMDs require frequent, parenteral administration (up to once daily). Due to the relatively early age of onset and chronic nature of MS, long-term treatment is often required. Self-injection is common, but performing regular injections can be stressful and physically demanding for many patients. Symptoms of the disease itself, such as poor manual dexterity and impaired cognitive function, can make self-injection more difficult. Other potential issues that may affect treatment acceptance and, thus, adherence include needle phobia, concerns about correct injection techniques, perceived lack of efficacy, treatment-related local and systemic side-effects and treatment fatigue [6-9]. Indeed, non-adherence to MS therapies is a common problem [10] with clinical consequences including reduced efficacy [11,12]. Patient education, a good doctor-patient relationship, management of patients' treatment expectations and accurate adherence monitoring are, therefore, essential to improve treatment adherence.

Mechanical, single-dose injection devices are available for most DMDs and can help patients to overcome injection-related issues [13,14]. A new electronic autoinjection device (RebiSmart™, Merck Serono S.A. - Geneva, Switzerland, an affiliate of Merck KGaA, Darmstadt, Germany) has been developed for the subcutaneous (sc) administration of IFN beta-1a supplied in a multi-dose cartridge. The new device is based on an existing device for administering growth hormone to children (easypod^®^, Merck Serono S.A. - Geneva) and has been adapted for use in patients with MS. The device has features that simplify the injection process and may help patients to overcome injection-related issues and improve treatment satisfaction, and may, therefore, increase adherence to treatment. These features include the convenience of the multi-dose cartridge, which holds one week's drug dosage; easy-to-understand, step-by-step instructions; an injection log to inform patients of their injection history; and adjustable comfort settings, which allow patients to tailor injections to improve comfort. Needle speed and injection speed, time and depth can all be changed by patients who experience discomfort upon injection or who are dissatisfied with the injection process, for example because a drop of liquid is seen on the skin when the needle is withdrawn.

This study was conducted to investigate the feasibility of using the device for the self-injection of sc IFN beta-1a, 44 mcg three times weekly (tiw), by patients with relapsing MS. To this end, patient-rated suitability of the device was assessed in a population of patients currently receiving IFN beta-1a via a single-dose autoinjection device and in healthcare professionals who trained patients in device use ('trainers'). The study also assessed patients' satisfaction with the injection process, patients' and trainers' evaluation of the device, local tolerability and overall safety.

## Methods

### Study design

The RebiSmart™ User Trial (study protocol 28733; NCT00735007) was a Phase IIIb, multicentre, 12-week, single-arm, open-label study carried out in 15 centres in Europe, the United States of America and Canada. The study was carried out in accordance with local clinical research regulations, approved by local ethics committees or institutional review boards (IRBs) and conformed to the World Medical Association Declaration of Helsinki (1996). A complete listing of the ethics committees that granted approval for the study is given below:

#### Canada

IRB services, Ontario; University of British Columbia Office of Research Services, Clinical Research Ethics Board, British Columbia.

#### Germany

Ethik-Kommission der Ärztekammer Hamburg; Ethik-Kommission bei der Landesärztekammer Baden-Württemberg, Stuttgart; Geschäftsstelle der Ethik-Kommission des Landes Berlin; Ethik-Kommission der Landesärztekammer Hessen.

#### Italy

Comitato di Etica per la Ricerca Biomedica, Università degli Studi "G. D'Annunzio", Azienda Sanitaria Locale - Chieti; Comitato Etico Azienda Policlinico Umberto I, Roma.

#### Spain

Secretaría Administrativa del Comité Etico de Investigacion Clinica, Hospital Universitario de Bellvitge, Barcelona; Comité Ético de Investigación Clínica, Hospital Clínico San Carlos, Madrid; Comité Ético de Investigación Clínica, Passeig Vall d'Hebrón, Barcelona.

#### Sweden

Regionala Etikprövningsnämnden i Lund.

#### USA

Chesapeake Research Review, Inc. Columbia, MD.

### Patients

Patients aged 18-65 years with a confirmed diagnosis of relapsing MS (according to the revised McDonald criteria [15]) with disease duration of at least 3 months were eligible. Inclusion criteria required patients to be already receiving the new formulation of IFN beta-1a that is free from serum-derived products, 44 mcg sc tiw, administered using the Rebiject II™ injection device, and to have been consistently on therapy for at least 6 weeks prior to screening. Women could not be pregnant or breastfeeding, and had to be without child-bearing potential. Key exclusion criteria included receiving any other medications via injection on a regular basis during the week prior to the screening period or throughout the study, receiving any therapy for MS other than sc IFN beta-1a in the 12 months prior to study enrolment or during the study, receiving oral or systemic corticosteroids or adrenocorticotrophic hormone in the 30 days prior to Study Day 1 (SD1), inadequate liver function or bone marrow reserve, moderate or severe renal impairment, a history of chronic pain syndrome, or any visual or physical impairment that would preclude the patient from using the device.

### Treatment

Patients self-administered the new formulation of sc IFN beta-1a, 44 mcg tiw, in a multi-dose cartridge, using the new electronic device for 12 weeks. The baseline device 'comfort settings' were the same for all patients.

### Study procedures and assessments

Patients who met the eligibility criteria during a 2-week screening period and gave written informed consent were recruited to the 12-week study. Baseline was defined as the first day of study drug administration (SD1). At SD1, patients were taught to self-inject using the new device by an investigator or a trainer and also underwent a physical examination. A safety assessment was performed, collecting information on adverse events (AEs) including injection-site reactions (ISRs), and routine blood samples were taken for laboratory testing. Patients completed items 13 to 38 of the Multiple Sclerosis Treatment Concern Questionnaire (MSTCQ) before the first injection with the new device, and the Patient User Trial Questionnaire after the first injection. They also received a patient diary and training on the recording of information such as injections and AEs.

At Week 2, a telephone call was made by a nurse to monitor device use by the patient and to collect information on AEs and concomitant medications. Further assessments were made at Weeks 4, 8 and 12. The Patient User Trial Questionnaire was completed at Weeks 4 and 12. The MSTCQ was completed and safety data collected at Weeks 4, 8 and 12. Local tolerability following injection was assessed at the follow-up visits by direct questioning of the patient and assessment of patient diaries.

Trainers (defined as the person who presents the patient with the device, trains them in device use and follows up during the study) evaluated the device by completing the Trainer User Trial Questionnaire at SD1 and Week 12, giving feedback on topics that included device functionality and ease of use.

### Study endpoints

#### Primary endpoint

The primary endpoint was the proportion of patients with relapsing MS rating the new device as 'very suitable' or 'suitable' for self-injecting the new formulation of sc IFN beta-1a at the end of the 12-week treatment period.

#### Secondary endpoints

Secondary endpoints included the incidence of predefined ISRs leading to treatment discontinuation at the end of Week 12 and evaluation of the injection process, treatment differences, and overall injection issues at Weeks 4, 8 and 12 using the MSTCQ. This evaluation included MSTCQ subscale scores for 'flu-like' symptoms (FLS), ISRs, global side-effects (GSE), and the most important benefit of the device (at SD1 and Weeks 4, 8 and 12). Injection pain was assessed using the short-form McGill Pain Questionnaire, visual analogue scale (VAS), and rating of pain (at SD1 and Weeks 4, 8 and 12). Overall evaluation of the device was based on responses from the Patient and Trainer User Trial Questionnaires (at SD1 and Weeks 4 and 12).

#### Safety endpoints

Safety endpoints included the incidence of treatment-emergent AEs, including serious AEs and abnormal laboratory parameters. According to the monitoring convention, only ISRs leading to treatment discontinuation were recorded as AEs. Other reactions at the injection site relating to sc IFN beta-1a administered using the new device were recorded on the local tolerability form as part of regular physician follow-up.

### Analysis populations

The intent-to-treat (ITT) population comprised all patients who had received at least one injection using the device and, for the primary endpoint, had a valid baseline and Week 12 assessment of device use. The per-protocol (PP) population was as for the ITT population, but excluded patients with major protocol violations. The safety population included all patients with follow-up safety data who had received at least one administration of study medication.

### Statistical analyses

For the primary endpoint, a sample of 100 patients was considered satisfactory to test the functionality and suitability of the new device. For the secondary endpoint (proportion of patients experiencing ISRs at the end of 12 weeks of treatment), a sample of 100 patients was required to determine a proportion of 28% (based on a post hoc analysis of data from a previous study) with a precision of 8-9%, using a 95% confidence interval (CI).

Descriptive statistics were calculated for continuous and categorical variables. No formal comparisons of data over time were performed. For the primary endpoint, the percentage of patients and corresponding exact 95% CIs (based on the Clopper-Pearson formula) were calculated; descriptive and inferential methods were used for secondary endpoints.

The primary endpoint was analysed only for patients with complete assessments at baseline and Week 12. A sensitivity analysis was conducted on the primary endpoint for the ITT population in which patients with missing data were assigned to the worst suitability category ('not at all suitable'), thereby including them in the denominator for the calculation. MSTCQ items related to FLS, ISRs and GSE subscales were imputed as stated in the MSTCQ scoring manual. Other missing data, including the primary endpoint, were not imputed.

## Results

### Patient disposition

Of the 108 patients screened, 106 were enrolled (ITT population). The two patients who failed screening did not meet the eligibility criteria. Of the 106 enrolled patients, 101 (95.3%) completed the 12 weeks of on-study treatment. Four of the five patients who withdrew from treatment did so between SD1 and Week 4. Reasons for premature treatment withdrawal were AEs (four patients) and protocol violation (one patient). Ten patients (9.4%) had at least one major protocol deviation and were excluded from the PP population. Major protocol deviations were: less than 75% compliance during the study (four patients; 3.8%); missing the Week 12 Patient User Trial Questionnaire (four patients; 3.8%); and inclusion criteria not met (two patients; 1.9%).

### Patient demographics

Baseline patient demographics are shown in Table 1. Consistent sc IFN beta-1a therapy for at least 6 weeks prior to screening was a requirement of study entry and, notably, 86 of the 106 enrolled patients (81.1%) had been taking sc IFN beta-1a for at least 1 year before starting the study.

**Table 1:** Baseline patient demographics, disease characteristics and treatment history.

Characteristic		Intent-to-treat population
Age, years	n (missing)	106 (0)
	Mean (SD)	41.7 (9.3)
	Median (range)	40.2 (23-63)
Sex, n (%)	Male	41 (38.7)
	Female	65 (61.3)
Race, n (%)	White	103 (97.2)
	Black	1 (0.9)
	Other	2 (1.9)
IFN beta-1a (sc) treatment before switching to new formulation of IFN beta-1a (sc)	n (missing)	106 (0)
	Yes	97 (91.5)
	No	9 (8.5)
Time from first IFN beta-1a (sc) treatment, years	n (missing)	97 (0)
	Mean (SD)	3.76 (2.54)
	Median (range)	3.20 (0.4-14.8)
Time from first IFN beta-1a (sc) treatment, n (%)	<6 months	3 (3.1)
	6 months-1 year	8 (8.2)
	1-2 years	16 (16.5)
	>2 years	70 (72.2)
Time from first treatment with new formulation of IFN beta-1a (sc), years	n (missing)	105 (1)
	Mean (SD)	0.73 (0.38)
	Median (range)	0.61 (0.2-1.4)
EDSS score	n (missing)	106 (0)
	Mean (SD)	2.2 (1.57)
	Median (range)	2.0 (0.0-6.5)
EDSS category, n (%)	0-<1	11 (10.4)
	1-<2	34 (32.1)
	1-<3	33 (31.1)
	3-<4	13 (12.3)
	4-<5	6 (5.7)
	5-<6	1 (0.9)
	≥6	8 (7.5)

EDSS, Expanded Disability Status Scale; IFN, interferon; sc, subcutaneous; SD, standard deviation.

### Trainer population

The trainer population comprised one trainer from each participating centre (i.e. 15 trainers in total).

### Suitability of the device for self-injection of sc IFN beta-1a

**Figure 1:**
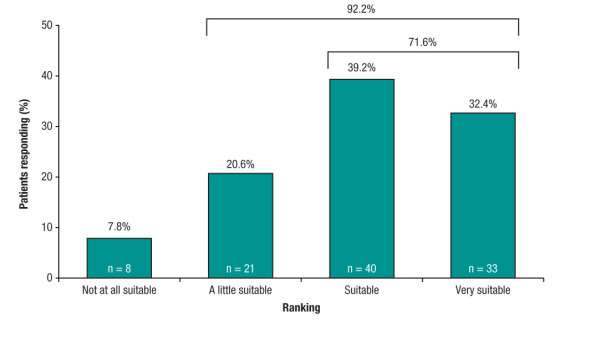
**Patient-rated suitability of the new device for self-injection of the new formulation of subcutaneous interferon beta-1a at Week 12: intent-to-treat population (n = 102, 4 missing)**.

At the end of Week 12, 71.6% (95% CI: 61.8%, 80.1%; 73/102) of patients found the new device to be 'very suitable' or 'suitable' for self-injection of sc IFN beta-1a (primary endpoint). Furthermore, 92.2% of patients reported some degree of suitability ('very suitable', 'suitable' or 'a little suitable'). Only 7.8% found the new device to be 'not at all suitable' for self-injection (Figure 1).

Sensitivity analyses, in which imputation of missing values was conducted by assigning patients with missing data to the worst suitability category ('not at all suitable') supported this finding: 68.9% (95% CI: 59.1%, 77.5%; 73/106) of patients found the new device to be 'very suitable' or 'suitable' for self-injection at Week 12.

### Evaluation of the injection process

The study was not designed to compare the new device with patients' previous experience of self-injection methods. However, certain items of the MSTCQ (administered at 4-week intervals) do assess changes in patients' experience of the injection process by recording feedback on injections in the last 4 weeks. For items 24 to 33 on the MSTCQ at Weeks 4, 8 and 12, the majority of patients reported that the process of performing injections with the new device over the previous 4 weeks (e.g. preparation, clean up, making injections, injection-related pain, time taken, ease of use of the system, FLS, and occurrence of injection reactions) was equivalent to, or compared favourably with, their previous experience of self-injection (Additional file 1).

Only minor changes were seen over time in parameters relating to the procedure of performing injections with the new device. For most of these parameters, the proportions of patients reporting their experience over the last 4 weeks as being at least equivalent to their previous experience with this device were slightly higher at Week 12 than at Week 4. Interestingly, the proportion of patients reporting that the injection procedure took less or about the same time increased from 56.3% at Week 4 to 76.2% at Week 12. Most patients found local injection reactions and pain (as assessed using the MSTCQ) to be equivalent or less problematic than previously, and there was little change in these measures over the duration of the study. At both Weeks 4 and 12, patients reported no change in overall injection issues and side-effects in the previous 4 weeks (data not shown).

### Overall device evaluation

**Figure 2:**
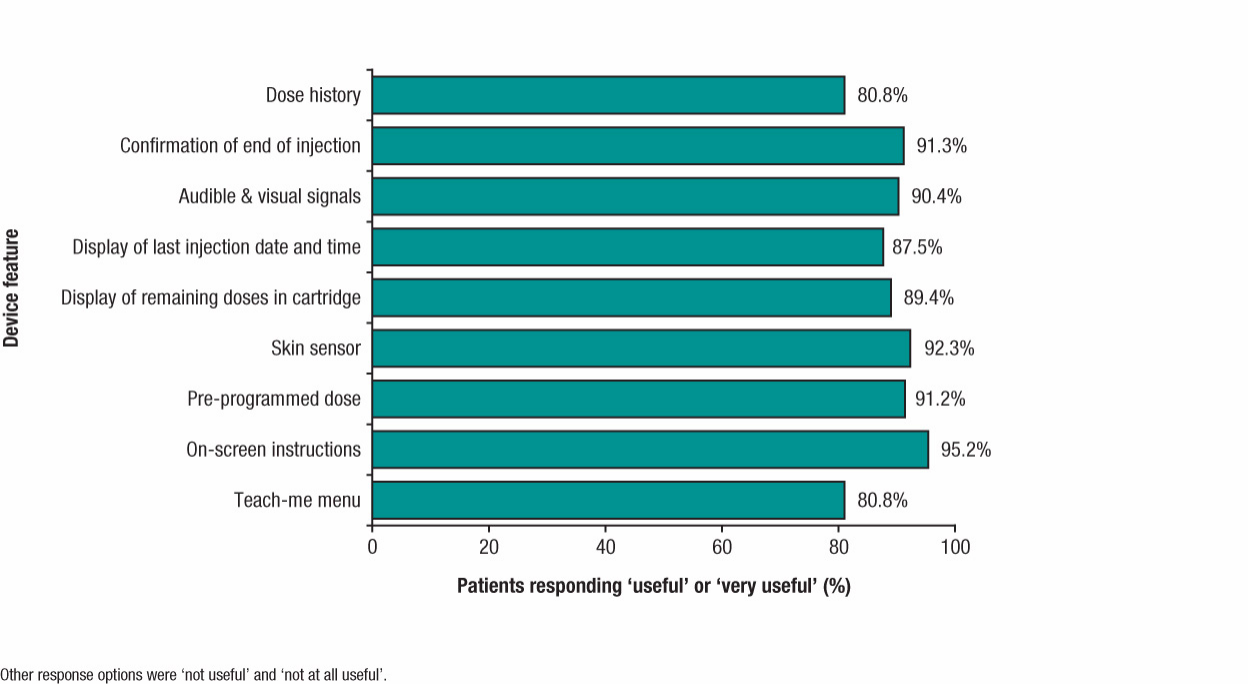
**Proportion of patients reporting device features as 'useful' or 'very useful' at Week 12 (n = 104 except for 'pre-programmed dose' [n = 103])**.

At Week 12, each of the device features (display of remaining doses in cartridge, display of last injection date and time, audible and visual signals, confirmation of end of injection, dose history, teach-me menu, on-screen instructions, pre-programmed dose, and skin sensor) was rated as 'useful' or 'very useful' by over 80% of patients; ratings for each feature are show in Figure 2. Further, 95.2% (99/104) of patients rated the use of device functions, generally, as 'very easy' or 'easy'. Each of the device actions (needle attachment, injection, needle detachment, cartridge change) was rated as 'easy' or 'very easy' to perform by over 80% of patients. Based on their experience, most patients rated 'overall convenience' to be the most important benefit of the new device (from a predefined list of responses) at all time points, reported by 63 of 85 respondents (74.1%) at Week 12.

At the end of Week 12, 71.2% (74/104) of patients rated the new device equal to or more convenient than their current device and 60.2% (62/103) of patients reported that they would prefer to keep the new device than revert to using their previous device.

All 15 trainers (100%) rated the device and device functions as 'easy' or 'very easy' to use.

### Comfort settings

By Week 12, 60.6% (63/104) of patients had changed the comfort settings on the device from baseline. For each comfort setting (needle speed, and injection speed, depth and time), a range of different settings were used by the 63 patients who changed these during the study.

### Tolerability of sc IFN beta-1a administered using the new device

**Figure 3:**
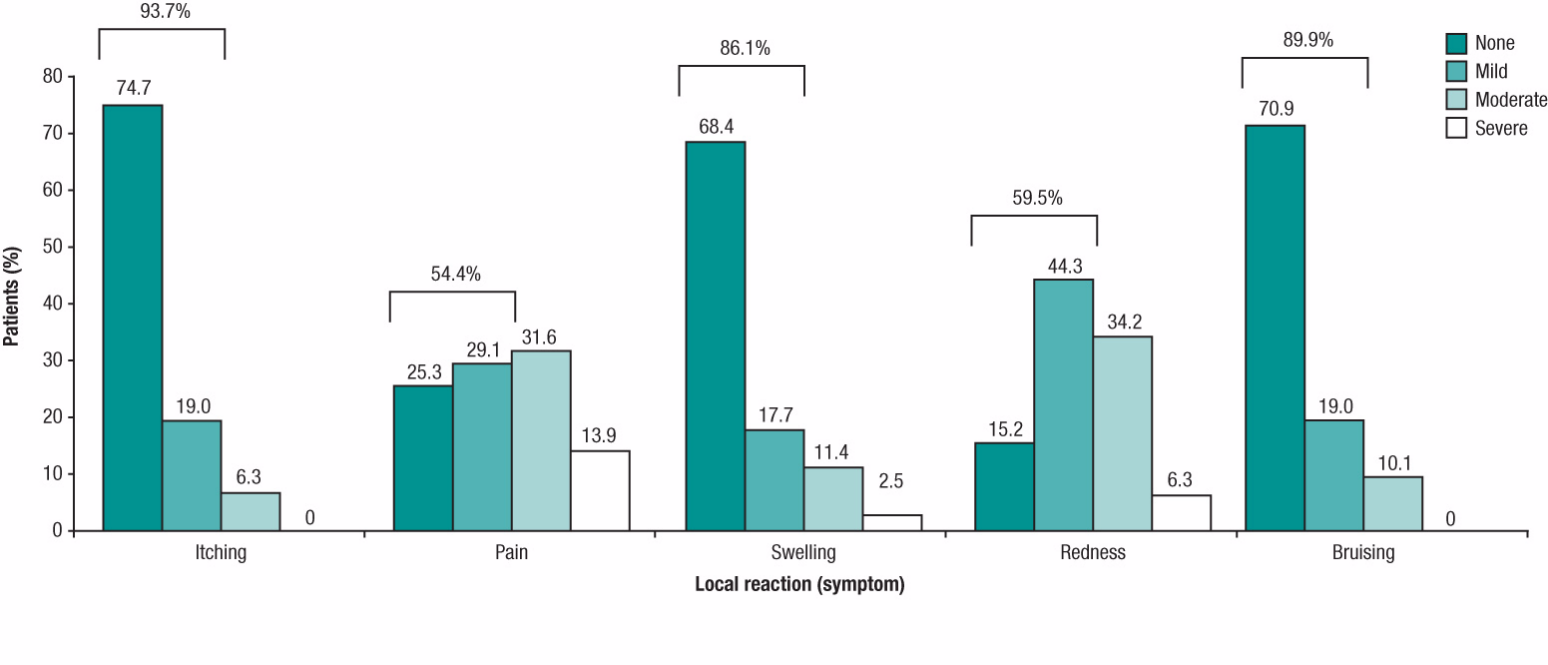
**Local tolerability of injections during 12-week study period (safety population, n = 106)**.

Local reactions at the injection site following self-injection (pain, swelling, redness or bruising), as reported by patients during follow-up visits and recorded by the physician on the local tolerability report form, occurred in 79 (74.5%) patients over the 12 weeks. Most reactions were considered mild or moderate in severity (Figure 3). Minimal changes in side-effects relating to ISRs in the previous 4 weeks (assessed via the MSTCQ) were reported by patients between SD1, which assessed the period when patients were using their previous injection device, and Week 12, which assessed their experience with the new device (Table 2). Similarly, there was little change over time in MSTCQ side-effect scores relating to FLS and GSE in the previous 4 weeks between SD1 and Week 12 (Table 2).

**Table 2:** MSTCQ scores for subscales relating to injection-site reactions (ISRs), 'flu-like' symptoms (FLS), and global side-effects (GSE), by 4-week interval.

		ISR score*		FLS score*		GSE score*	
		
Time point		Observed	Change	Observed	Change	Observed	Change
Study Day 1	n (missing)	106 (0)	-	106 (0)	-	106 (0)	-
	Mean (SD)	11.1 (3.3)	-	9.5 (3.7)	-	4.8 (1.7)	-
	Median	11.0	-	10.0	-	4.0	-
	(range)	(4-19)		(4-17)		(3-10)	
Week 4	n (missing)	103 (3)	103 (3)	103 (3)	103 (3)	103 (3)	103 (3)
	Mean (SD)	11.4 (3.2)	0.3 (3.3)	8.8 (3.9)	-0.7 (3.3)	5.5 (2.4)	0.7 (2.3)
	Median	12.0	0.0	9.0	0.0	5.0	0.0
	(range)	(4-18)	(-10-9)	(4-17)	(-9-10)	(3-13)	(-4-9)
Week 8	n (missing)	100 (6)	100 (6)	100 (6)	100 (6)	100 (6)	100 (6)
	Mean (SD)	11.3 (3.5)	0.2 (3.6)	8.8 (3.8)	-0.8 (3.0)	5.2 (2.2)	0.5 (2.2)
	Median	12.0	0.0	9.0	0.0	5.0	0.0
	(range)	(4-18)	(-11-8)	(4-17)	(-11-8)	(3-12)	(-4-7)
Week 12/early termination	n (missing)	106 (0)	106 (0)	106 (0)	106 (0)	106 (0)	106 (0)
	Mean (SD)	11.4 (3.4)	0.3 (3.4)	9.0 (3.8)	-0.5 (3.3)	5.6 (2.6)	0.8 (2.5)
	Median	12.0	0.0	9.0	0.0	5.0	0.0
	(range)	(4-19)	(-11-8)	(4-18)	(-7-11)	(3-14)	(-4-9)

MSTCQ, Multiple Sclerosis Treatment Concern Questionnaire; SD, standard deviation.

*Maximum score = 20 for ISR and FLS, 15 for GSE; higher scores indicate greater negative impact. Further information on the use of the MSTCQ to assess satisfaction with injection systems for subcutaneous interferon beta-1a can be found in [13].

Pain on injection, as assessed using the Pain Rating Grade and Pain Rating Scale (VAS), did not differ between Weeks 4 and 12 (data not shown). Over half of the patients reported no or only mild pain on injection on the Pain Rating Grade from Week 4 (61.4%; 62/101) to Week 12 (56.6%; 60/106). There were no clear differences between SD1 and Week 12 in injection-related pain, as assessed by the McGill Pain Questionnaire. Most pain symptoms were reported as absent by more than 70% of patients at both SD1 and Week 12.

### Safety

No new or unexpected safety issues associated with exposure to sc IFN beta-1a were observed during this study. Serious AEs occurred in three (2.8%) patients. AEs are summarized in Table 3. Prespecified ISRs leading to treatment withdrawal occurred in four (3.8%) patients. Some patients discontinued treatment because they had experienced more than one AE. Four patients (3.8%) experienced AEs that led to permanent, premature discontinuation of treatment: injection-site pain (n = 3), injection-site erythema (n = 2), injection-site irritation (n = 2) and influenza-like illness (n = 1).

**Table 3:** Adverse events (AEs) reported during the 12-week study (safety population).

	Patients, n (%) (n = 106)	Events, n (%) (n = 193)
*Treatment-emergent AE*		
*Any*	*67 (63.2)*	*193 (100.0)*
Influenza-like illness*	22 (20.8)	54 (28.0)
Headache*	12 (11.3)	25 (13.0)
*Severity of treatment-emergent AEs*		
Mild	43 (40.6)	96 (49.7)
Moderate	35 (33.0)	73 (37.8)
Severe	14 (13.2)	24 (12.4)
*Prespecified AEs*		
*Any*	*32 (30.2)*	*74 (38.3)*
'Flu-like' symptoms	22 (20.8)	54 (28.0)
Cytopenia	5 (4.7)	9 (4.7)
Injection-site reactions	4 (3.8)	8 (4.1)
Hypersensitivity	2 (1.9)	3 (1.6)
Rash (pruritus)	1 (0.9)	2 (1.0)
*Serious AEs*		
*Any*	*3 (2.8)*	*6 (3.1)*
Myocardial infarction	1 (0.9)	1 (0.5)
Adverse drug reaction	1 (0.9)	1 (0.5)
Biliary colic	1 (0.9)	1 (0.5)
Breast infection	1 (0.9)	1 (0.5)
Seroma	1 (0.9)	1 (0.5)
Hypertensive crisis	1 (0.9)	1 (0.5)

*Most common (occurring in >10% of patients).

## Discussion

The RebiSmart™ User Trial investigated the feasibility of the new electronic injection device to self-inject sc IFN beta-1a in patients with relapsing MS, primarily by assessing patient-rated suitability of the device and evaluation of the device and the injection process. Over 90% of patients in this study reported some degree of suitability and 71.6% considered the device 'suitable' or 'very suitable' for this purpose. The sensitivity analysis, which considered the worst-case scenario by assigning missing patients to the 'not at all suitable' category, supported this finding.

No new safety concerns were raised in this study; sc IFN beta-1a was generally well tolerated, with the AEs being consistent with the known safety profile of this drug [5,16]. As expected, there was little difference in the incidences of FLS and local ISRs throughout the study, indicating that AEs were largely unaltered by a change in injection device. Importantly, overall injection issues did not change notably, and side-effects relating to sc IFN beta-1a self-administration using the new device generally remained stable over the 12-week study.

Injection devices have been shown to reduce ISRs [14], at least partly by ensuring correct injection technique. As ISRs are probably related more to the study drug than the device, the little or no change in local tolerability seen over the course of this study was as expected. Notably only four patients (3.8%) experienced prespecified ISRs as an AE, all of which occurred within the first 4 weeks of the study. Patient-reported local reactions at the injection site, as recorded by physicians on the local tolerability report form (including redness, pain, bruising or itching), occurred in 74.5% of patients over the 12 weeks. However, 75.2% stated that ISRs did not interfere at all with daily work and activities (data not shown).

Injection-related pain, recorded through various assessment tools, generally did not change over the course of the study. Pain on injection was given as a reason for changing comfort settings, but at the time of the trial there was no available information on optimum comfort settings in different patient groups. As it was not possible to provide patients with guidance on setting algorithms, it is presumed that patients had to experiment with different settings before finding their most comfortable injection settings. Over such a short (12-week) study, without guidelines, patients may not have felt confident in changing the settings despite being trained in this device function, which may explain why 40% made no changes at all. Thus, patients may not have found their optimum settings. Further studies are needed to determine optimum comfort settings in different patients.

As with other ISRs, pain is probably not related to the device. At the time of the study, refrigerated storage of the new formulation in the multi-dose cartridge was necessary, and injection of the drug that was below room temperature may have resulted in ISRs, including pain. It is possible that patients familiar with the single-dose syringe may have underestimated the time needed to warm the larger volume of drug in the multi-dose cartridge within the device to room temperature. In addition, while patients were advised to keep the solution out of the fridge for 1 hour prior to injection, this may have been insufficient for the solution to reach room temperature in some situations. Indeed, it is possible that no discontinuations due to AEs occurred after 4 weeks at least partly because patients were more practiced in the entire injection process, including the time required to warm the drug to room temperature in their surroundings. However, as detailed information on injection preparation was not formally collected, we cannot confirm that injection of cold drug contributed to the relatively high incidence of ISRs seen in this study. Correct injection preparation can be addressed through patient education and support, but it should be noted that since completing the study, approval has been granted for room temperature storage of the multi-dose cartridge for up to 2 weeks [17], making refrigeration of the pre-loaded device no longer necessary.

The positive findings of this study must be considered in the context of the study limitations. Although the Patient User Trial Questionnaire was used previously to assess the suitability of a similar device in children and adolescents requiring growth hormone therapy, this questionnaire has not yet been validated in an adult, MS population. Furthermore, patients may have found the assessment of 'device suitability' difficult to interpret, particularly with respect to differences between 'suitable' and 'a little suitable'. Problems with interpretation may have been confounded by translation of 'suitability' into different languages. The inclusion of a defined patient subgroup only may also have influenced results.

The finding that the majority of this population of patients with relapsing MS considered the device to be suitable for their use is particularly encouraging considering that over 80% had been receiving IFN beta-1a therapy for over 1 year at study entry (the mean time since starting sc IFN beta-1a treatment was 3.76 years), and all patients had previous experience with autoinjector technologies. It is possible that changes to injection practice might result in lower satisfaction with treatment in patients who are already comfortable with their existing administration method, particularly over the relatively short duration of this 12-week study. Therefore, this study population may be an appropriately stringent group of patients in which to assess responses to a new injection device.

It is reasonable to speculate that patients who are naïve to, or who have discontinued treatment due to, injection-related issues may perceive a greater benefit in a new device. Nonetheless, even in the current study population, the device was generally well received, with over 80% of patients rating the various device features as 'useful' or 'very useful' and more than 95% considering the device functions as 'easy' or 'very easy' to perform. Indeed, at the end of the study, 60.2% of patients stated that they would prefer to continue using the new device than to return to their previous device. This proportion might seem low considering that very few patients discontinued treatment over the study period and over half considered the device to be more convenient than their previous device. However, it is possible that this finding may reflect a desire by a proportion of patients to return to an injection device with which they had experience outside of the clinical trial environment. In addition, the findings of this 12-week study suggested that a 'learning curve' existed even over this relatively short time-period, with patients becoming more adept at using the device over time. The proportion of patients who found overall convenience to be the most important benefit of the device increased from 42.2% at SD1 to 74.1% at Week 12, and the percentage of patients who reported that a shorter or similar amount of time was needed for the procedure than previously rose from 56.3% at Week 4 to 76.2% at Week 12. Responses for other MSTCQ items relating to performing injections also tended to become more positive over time, suggesting that patients were becoming more proficient in preparing and performing injections with the new device. The proportion of patients wishing to continue using the new device may therefore have increased with prolonged use.

Despite patient awareness of the potentially debilitating nature of MS, long-term adherence to therapy is known to be poor [10]. Failure to adhere to prescribed treatment can have several consequences, including reduced efficacy, increased healthcare costs and poor long-term health outcomes. Among the many factors that can lead to treatment discontinuation [9], anxiety and concerns over self-injection are strong predictors of treatment discontinuation after 6 months [8]. Adherence to therapy [18,19] and shorter treatment gaps [11] have been shown to decrease the risk of severe relapse in patients with MS receiving DMD therapy. Improving treatment adherence is, therefore, an important goal.

Delivery devices have the potential to help patients overcome injection-related problems, such as ISRs and fear of incorrect injection technique, which may improve adherence. Indeed, the use of injection devices can substantially reduce ISRs compared with injection with syringe alone [14], and improvements in device technology have been shown to reduce ISRs and pain on injection, and improve treatment satisfaction [13]. In addition to shielding the needle from view and improved convenience, which are benefits common to all currently available injection devices, the new electronic injection device has several specific additional features that may further benefit patients. The ability to adjust injection comfort settings may encourage patients to experiment with new settings and customize these to their personal preferences. This function may enable patients to continue therapy where they may previously have chosen to stop treatment. Additionally, over 90% of patients considered confirmation of successful injection to be useful in providing reassurance that they had administered their injections correctly. The multi-dose cartridge contains 1 week's worth of medication, which reduces the frequency of device loading. The novel dosing log informs the patient that an injection is due and serves as a reminder of treatment history, which may be particularly useful to patients with cognitive impairment. The log also enables adherence to MS treatment to be recorded accurately and objectively for the first time. In contrast, all previous measures of adherence have relied on subjective, often retrospective, patient reporting, which can give inaccurate results. Dosing history data can also be downloaded, linked to electronic patient records and reviewed by physicians, and thus may promote an open dialogue between patient and neurologist regarding treatment adherence, although this was not assessed in this study. Furthermore, as adherence to treatment can influence outcomes, assessment of efficacy in both trials and in the clinic may be confounded by the patient's subjective reporting of adherence. Hence, a device that can provide an objective measure of adherence may also be a critical tool in assessing the true relationship of treatment to patient outcomes. Treatment adherence in patients using the device is currently being assessed in an ongoing study being carried out in Canada (Study EMR701068_520). In addition, it may be interesting to compare treatment adherence and disease outcomes in patients using the new device and in those using other delivery methods.

## Conclusions

This study gave patients with relapsing MS who had been consistently self-administering sc IFN beta-1a for at least 6 weeks prior to the study the opportunity to test and assess the suitability of a new electronic injection device for self-injection of their current therapy. The vast majority of patients in the study considered the new device to be suitable for the self-administration of sc IFN beta-1a for the treatment of relapsing MS (over 90% of patients reported some degree of suitability). In addition, the device was found to be easy to use and to have features considered useful by most patients. Most patients also found using the new device either equivalent to or better than their previous experience for a range of measures relating to the injection procedure, and over half wanted to continue using the device at the end of the study. Based on these findings, it is reasonable to predict that the device may also be suitable for those who are not satisfied with existing administration methods or those new to DMD therapy. Furthermore, the benefits and device features may help patients to adhere to their prescribed therapy and provide a basis for discussions between patients and physicians in order to optimize adherence and so maximize outcomes with chronic treatment for MS.

## Competing interests

**AL **has received honoraria or research grants from: Biogen Dompè, Sanofi Aventis, Merck Serono S.A. - Geneva and Bayer Schering, and is involved in clinical trials of the same companies, plus GlaxoSmithKline and Teva Neuroscience.

**TA **has received honoraria for lecturing and consulting services, travel expenses for attending meetings and financial support for research from Bayer Schering, Biogen Idec, Sanofi Aventis, Teva Neurosciences and Merck Serono S.A. - Geneva.

**BB **has received honoraria from: Biogen Idec, GlaxoSmithKline, Janssen-Cilag, Merck Serono S.A. - Geneva; and has been involved in clinical trials with the same companies as well as Pfizer.

**BS **has received consulting and/or speaker honoraria from Bayer, Biogen Idec, EMD Serono, Inc., Novartis, Pfizer and Teva Neuroscience; and research funding from Biogen Idec, EMD Serono, Genzyme and Novartis.

**ML **has no competing interests to declare.

**VD **has received honoraria from: Biogen Idec, Merck Serono S.A. - Geneva, Bayer and Teva Neuroscience.

**EV **and **PC **are employees of Merck Serono S.A. - Geneva, Switzerland, an affiliate of Merck KGaA, Darmstadt, Germany.

## Authors' contributions

All the authors contributed to the manuscript and have read and approved the final version. VD^†^ developed the first draft of the manuscript. EV developed the study protocol and approved the statistical analysis plan. PC developed the statistical analysis plan and performed the statistical analysis.

## Pre-publication history

The pre-publication history for this paper can be accessed here:

http://www.biomedcentral.com/1471-2377/10/28/prepub

## Supplementary Material

Additional file 1 **Supplemental Table**. Patient assessment of the injection process in the previous 4 weeks; intent-to-treat population (n = 106)*. Click here for file
