# Supplementary material for: Patient-rated suitability of a novel electronic device for self-injection of subcutaneous interferon beta-1a in relapsing multiple sclerosis: an international, single-arm, multicentre, Phase IIIb study
Source: BMC Neurol. 2010 Apr 30;10:28. doi: 10.1186/1471-2377-10-28 (PMC2877661; doi:10.1186/1471-2377-10-28)
Supplement: Additional file 1 — Supplemental Table. Patient assessment of the injection process in the previous 4 weeks; intent-to-treat population (n = 106)*. [file 1471-2377-10-28-S1.DOC]

**Supplementary Table**

**Patient assessment of the injection process in the previous 4 weeks; intent-to-treat population (n = 106)***

| **MSTCQ Item** | **Response** | **n (%)** | | |
| --- | --- | --- | --- | --- |
|  |  | **Week 4** | **Week 8** | **Week 12** |
| 24. Is preparation for injections more or less bothersome now? | Much less | 36 (35.0) | 34 (34.0) | 36 (34.0) |
|  | Somewhat less | 19 (18.4) | 25 (25.0) | 16 (15.1) |
|  | About the same | 19 (18.4) | 26 (26.0) | 39 (36.8) |
|  | Somewhat more | 25 (24.3) | 14 (14.0) | 13 (12.3) |
|  | Much more | 4 (3.9) | 1 (1.0) | 2 (1.9) |
| 25. Is clean up after injections more or less bothersome now? | Much less | 40 (38.8) | 34 (34.0) | 35 (33.0) |
|  | Somewhat less | 15 (14.6) | 17 (17.0) | 12 (11.3) |
|  | About the same | 35 (34.0) | 42 (42.0) | 49 (46.2) |
|  | Somewhat more | 10 (9.7) | 6 (6.0) | 8 (7.5) |
|  | Much more | 3 (2.9) | 1 (1.0) | 2 (1.9) |
| 26. Is making self-injections more or less difficult now? | Much less | 36 (35.0) | 30 (30.0) | 30 (28.3) |
|  | Somewhat less | 24 (23.3) | 23 (23.0) | 22 (20.8) |
|  | About the same | 20 (19.4) | 36 (36.0) | 36 (34.0) |
|  | Somewhat more | 20 (19.4) | 9 (9.0) | 14 (13.2) |
|  | Much more | 3 (2.9) | 2 (2.0) | 4 (3.8) |
| 27. Are injections more or less painful now? | Much less | 13 (12.6) | 14 (14.0) | 16 (15.1) |
|  | Somewhat less | 21 (20.4) | 14 (14.0) | 14 (13.2) |
|  | About the same | 30 (29.1) | 35 (35.0) | 40 (37.7) |
|  | Somewhat more | 25 (24.3) | 24 (24.0) | 22 (20.8) |
|  | Much more | 14 (13.6) | 13 (13.0) | 14 (13.2) |
| 28. Is the amount of time needed for the whole procedure longer or shorter now? | Much shorter | 17 (16.5) | 19 (19.0) | 24 (22.9) |
|  | Somewhat shorter | 23 (22.3) | 22 (22.0) | 17 (16.2) |
|  | About the same | 18 (17.5) | 31 (31.0) | 39 (37.1) |
|  | Somewhat longer | 33 (32.0) | 24 (24.0) | 19 (18.1) |
|  | Much longer | 12 (11.7) | 4 (4.0) | 6 (5.7) |
| 29. Overall, was it easier to use the injection system? | Much easier | 27 (26.2) | 35 (35.0) | 34 (32.1) |
|  | Somewhat easier | 37 (35.9) | 23 (23.0) | 20 (18.9) |
|  | About the same | 17 (16.5) | 29 (29.0) | 34 (32.1) |
|  | Somewhat less easy | 17 (16.5) | 10 (10.0) | 14 (13.2) |
|  | Much less easy | 5 (4.9) | 3 (3.0) | 4 (3.8) |
| 30. Do injection reactions occur more or less often now? | Much less | 12 (11.7) | 12 (12.0) | 10 (9.4) |
|  | Somewhat less | 15 (14.6) | 13 (13.0) | 13 (12.3) |
|  | About the same | 45 (43.7) | 43 (43.0) | 49 (46.2) |
|  | Somewhat more | 16 (15.5) | 17 (17.0) | 18 (17.0) |
|  | Much more | 15 (14.6) | 15 (15.0) | 16 (15.1) |
| 31. Are injection reactions more or less bothersome now? | Much less | 6 (5.9) | 10 (9.9) | 10 (9.4) |
|  | Somewhat less | 18 (17.6) | 10 (9.9) | 13 (12.3) |
|  | About the same | 53 (52.0) | 57 (56.4) | 55 (51.9) |
|  | Somewhat more | 18 (17.6) | 19 (18.8) | 18 (17.0) |
|  | Much more | 7 (6.9) | 5 (5.0) | 10 (9.4) |
| 32. Do ‘flu-like’ side-effects from the medication (tiredness, fever) occur more or less often now? | Much less | 15 (15.0) | 13 (13.0) | 13 (12.4) |
|  | Somewhat less | 10 (10.0) | 12 (12.0) | 6 (5.7) |
|  | About the same | 57 (57.0) | 63 (63.0) | 75 (71.4) |
|  | Somewhat more | 14 (14.0) | 10 (10.0) | 8 (7.6) |
|  | Much more | 4 (4.0) | 2 (2.0) | 3 (2.9) |
| 33. Are ‘flu-like’ side-effects from the medication (tiredness, fever) worse or less bothersome now? | Much less | 14 (14.0) | 14 (14.1) | 12 (11.4) |
|  | Somewhat less | 7 (7.0) | 6 (6.1) | 6 (5.7) |
|  | About the same | 65 (65.0) | 71 (71.7) | 78 (74.3) |
|  | Somewhat more | 11 (11.0) | 6 (6.1) | 5 (4.8) |
|  | Much more | 3 (3.0) | 2 (2.0) | 4 (3.8) |

MSTCQ, Multiple Sclerosis Treatment Concern Questionnaire.

*Patient numbers may not total 106 as some patients did not complete all items.
